# Correction to: Elevated energy costs of biomass production in mitochondrial respiration-deficient *Saccharomyces cerevisiae*

**DOI:** 10.1093/femsyr/foad015

**Published:** 2023-03-28

This is a correction to: Pranas Grigaitis, Samira L van den Bogaard, Bas Teusink, Elevated energy costs of biomass production in mitochondrial respiration-deficient *Saccharomyces cerevisiae, FEMS Yeast Research*, Volume 23, 2023, foad008, https://doi.org/10.1093/femsyr/foad008

In the originally published version of this manuscript, the title was incorrectly written as ‘Elevated energy costs of biomass production in mitochondrial respiration-deficient *Saccharomyces cerevisia*’. The correct title is ‘Elevated energy costs of biomass production in mitochondrial respiration-deficient *Saccharomyces cerevisiae*’.

This error has now been corrected.

